# Maternal Mental Health, Child Distress and Family Strain During the COVID-19 Pandemic: Linking the Provincial Longitudinal Cohort with the COVID-19 Impact Survey Data in Canada.

**DOI:** 10.23889/ijpds.v7i3.1917

**Published:** 2022-08-25

**Authors:** Janelle Boram Lee, Henry Ntanda, Kharah Ross, Nicole Letourneau

**Affiliations:** 1 University of Calgary; 2 Athabasca University

## Objectives

To understand the impact of the COVID-19 pandemic on families in Canada by specifically examining the relationship between maternal mental distress (MD), child distress (CD) and family strain (FS) trends over time. We linked the Alberta Pregnancy Outcomes and Nutrition (APrON) longitudinal cohort data and COVID-19 Impact Survey (CIS).

## Approach

Three waves of CIS (March 2020 to July 2021), collected from APrON longitudinal cohort, were used. Demographic variables from APrON were linked with CIS. Mothers’ depression, anxiety, and/or stress scores were standardized separately for different symptoms, averaged at each wave, and combined as one maternal MD variable (low/medium/high). CD was measured across emotional, conduct, hyperactivity, and peer problem scales (low/high). FS was defined as COVID-19 straining family relationships, including partners, parent-child, and siblings (yes/no). Latent class analyses were performed to identify and categorize membership across the variables. To address the objective, multiple logistic regression models were conducted.

## Results

The sample consisted of 157 participants were included in the study; 19.1% reported FS during COVID-19. Three latent classes were formed for maternal MD: consistently low (36.9%), medium (44.0%), and high (19.1%) across the follow-up period. Two latent classes were formed for CD: consistently low (79.6%) and high (20.4%). When adjusted for COVID-19 related covariates (e.g., maternal worries about child’s well-being/education, family difficulty with childcare/schoolwork) and socioeconomic status, mothers with medium and high levels of maternal MD were at increased odds of experiencing FS during the COVID-19 pandemic compared to those with a low level of distress (medium aOR = 3.90[1.08, 14.03]; high aOR = 4.57[1.03, 20.25]). The adjusted association between child distress and FS was not statistically significant (aOR = 1.75[0.59, 5.20]).

## Conclusion

Understanding how MD could affect family strain is important as many families recover from the pandemic. More distressed individuals experience greater FS over time, suggesting this association as a chronic problem. Stakeholders should tailor support systems to longer-term, family-level interventions improving family relationships and maternal-child MHD impacted by COVID-19.

